# Peritoneal dialysis catheter insertion in patients with obesity: a cohort study and single centre approach to increasing uptake

**DOI:** 10.1186/s12882-025-04210-3

**Published:** 2025-07-01

**Authors:** Walker Andrew, Corr Michael, Conroy Daniel, McDaid James, McGrogan Damian, Fogarty Damian, O’Neil Stephen

**Affiliations:** 1 Regional Nephrology & Transplant Unit-Belfast Health and Social Care Trust, Belfast, UK; 2 Centre for Public Health, Institute of Clinical Sciences B, Royal Victoria Hospital, Belfast, BT12 6BA UK; 3 Interventional Radiology Department, Belfast Health and Social Care Trust, Belfast, UK; 4 Centre for Medical Education- Queen’s University Belfast, Belfast, UK

**Keywords:** Obesity, Peritoneal dialysis, Peritonitis, Catheter, Kidney replacement therapy

## Abstract

**Background:**

The global prevalence of obesity in patients with end-stage kidney disease requiring kidney replacement therapy is rising. While peritoneal dialysis (PD) offers advantages for many patients, its use in those with obesity has been historically limited due to concerns about catheter insertion-related complications, mechanical issues, and infection risk. This study aimed to evaluate PD catheter patency, infection rates, and modality outcomes in obese and non-obese patients to inform best practices and guide programme expansion.

**Methods:**

This single-region observational study analysed outcomes from 157 patients who underwent PD catheter insertion between 2020 and 2023 at the Northern Ireland Regional Nephrology and Transplant Centre. Patients were classified as obese (BMI ≥ 30, n = 44) or non-obese (BMI < 30, n = 113). Laparoscopic and percutaneous catheter insertion techniques were used, and primary outcomes included catheter patency (primary, primary assisted, secondary) and infection rates (exit site infection, tunnel infection, peritonitis) 1-year post-insertion. Kaplan-Meier survival analysis and descriptive statistics were applied to compare outcomes between groups.

**Results:**

Patency rates were high in both groups, with no significant differences: primary patency was 88% in non-obese patients and 80% in obese patients (p = 0.13). Similarly, primary assisted patency was 94% versus 89% (p = 0.26), and secondary patency was 96% versus 89% (p = 0.11). Patency and infection rates remained within standards set in ISPD guidelines for both groups, although obese patients showed a non-significant trend toward higher peritonitis rates (0.09). Transfer to haemodialysis occurred more frequently in the obese group (34% vs. 19%, p = 0.06). Mortality rates were comparable (9% non-obese, 6% obese).

**Conclusions:**

This study demonstrates that, with appropriate surgical techniques and periprocedural care, obese patients can achieve PD outcomes comparable to those of non-obese individuals. These findings challenge the traditional reluctance to offer PD to obese patients and advocate for the expansion of PD programs to include this growing demographic.

**Clinical Trial Number:**

Not Applicable

**Supplementary Information:**

The online version contains supplementary material available at 10.1186/s12882-025-04210-3.

## Background

The prevalence of obesity in patients with end stage kidney disease (ESKD) requiring kidney replacement therapy continues to rise globally [1, 2]. High rates of obesity in the general population are thought to be a major contributor to increasing levels of ESKD due to diabetic and hypertensive nephropathy [3]. This is true in lower and middle income nations where there are significant rises in obesity and ESKD [1–3]. Whilst patients living with chronic kidney disease (CKD) and ESKD may develop obesity from treatment effects such as immunosuppressive medications or fatigue/reduced exercise capacity due to living with a chronic illness [4].

Providing optimal kidney replacement therapy for individuals living with obesity can be challenging. Transplantation has increased risks of complications with many centres having Body Mass Index (BMI) cutoffs for transplant listing [5, 6]. Meanwhile, in haemodialysis (HD) the formation of definitive vascular access in obesity can be limited with an increased prevalence of central venous catheters used for dialysis in this population [7]. Peritoneal dialysis (PD) has traditionally been underutilised in patients with obesity partly due to reported catheter-related and mechanical complications, concerns around anaesthetic risk and reported higher rates of PD associated infection [8–10].

The primary challenge in providing PD access to obese patients lies in achieving an optimal balance between positioning the catheter tip within the pelvis for effective hydraulic function and locating the exit site in an environmentally favourable zone that the patient can easily see and maintain. Alongside the PD catheter insertion technique employed, determining the insertion site and selecting the most appropriate catheter length are crucial to attaining this balance, thereby ensuring the best possible outcomes in terms of minimising mechanical and infectious complications [8, 9]. The literature reporting PD catheter insertion outcomes in obesity is limited and the optimal catheter insertion approach remains controversial [8]. Whilst within the general PD population advanced laparoscopic PD catheter insertion techniques produce better functional outcomes, their specific role in PD access for the patient living with obesity remains to be fully elucidated [11–13].

Given the potential advantages for both individual patients and the wider healthcare system, many centres providing treatment for ESKD seek to expand their PD programmes [14, 15]. There may be particular benefits to increasing PD programmes in lower and middle income nations due to the decreased costs of PD over HD [14]. However, hesitancy regarding PD catheter insertion, potential mechanical and infective complications in obesity may limit this goal. Our centre, having traditionally utilised PD less than other UK based centres, has been attempting to increase our PD services for patients [16, 17]. The aim of this study was to compare the PD catheter insertion patency and associated mechanical and infective complications in those living with or without obesity.

## Methods

### Study design

This is a single-region observational study analysing data from a prospectively collected PD database. This study adheres to the STrengthening the Reporting of OBservational studies in Epidemiology (STROBE) reporting guidelines for the dissemination of observational research [18]. A completed checklist can be found in Appendix A.

### Study setting

This study was conducted in Northern Ireland (NI), a region of the United Kingdom with a population of 1.9 million spread over 14,130 km² (population density: 135/km²). The region has an annual incidence of approximately 205 patients (108 per population million) starting kidney replacement therapy across five hospital trust sites, each equipped with a HD unit, but with a single central transplant and renal surgical centre [16]. All PD catheter insertion procedures are performed at the Regional Nephrology and Transplant Centre.

### Ethical review

The Northern Ireland Peritoneal Dialysis Access Database and its use for research purposes has been ethically approved (REC 24/LO/06810). The requirement for individual consent was waived by the authorising Research Ethics Committee.

### Data collection and analysis

All patients who had a PD catheter inserted from 01/01/2020-31/12/2023 were included. Patients were followed up 1-year post insertion of the catheter up until 31/12/2024. Obesity was defined having a Body Mass Index (BMI) ≥ 30, noting its limitations as a measurement for obesity [8, 9]. Baseline characteristics collected included age, sex, primary renal disease, prior abdominal surgery, and modality at time of catheter insertion. Laboratory parameters, comorbidity scores, and formal nutritional status indicators were not routinely collected during the study period.

The primary outcome measured in the study was patency of the PD access. Primary patency was defined as a functioning PD catheter that did not require removal, replacement, or requirement for intervention because of flow dysfunction or drain pain [19]. In this study, catheter patency outcomes were classified using adapted vascular access terminology (primary, primary assisted, and secondary patency) to distinguish outcomes achieved without intervention, with intervention, and after catheter replacement respectively, providing a more granular view of catheter survival [17]. Primary assisted patency was defined as a functioning PD catheter that did require manipulation because of flow dysfunction or drain pain. Secondary patency was defined as a functioning PD catheter after the previous catheter needed to be replaced because of flow dysfunction or drain pain. Loss of patency is censored for death, transplant, infection, or transfer to HD for non-PD related reasons (patient preference, independent medical condition necessitating modality transfer) [19, 20]. Patency rates were compared to the benchmark set by the International Society for Peritoneal Dialysis (ISPD) guidelines on creating and maintaining PD access [19].

The secondary outcomes measured in this study were the 1-year post catheter insertion rates of exit site infection, tunnel infection and PD associated peritonitis. The ISPD guidelines for catheter related infections were used to define infection cases and the subsequently compared to maximum tolerated infection rates detailed in the guideline [21].

Kidney replacement modality transfer and mortality data were also collected and analysed for observed differences between both the non-obese and obese cohorts.

### Statistical Analysis

Demographic and clinical details were compared by T-test with statistical significance defined as p < 0.05. Time-to-event survival analysis was used to evaluate primary, primary assisted, and secondary patency of peritoneal dialysis catheters. A Kaplan–Meier curve was then generated to graphically display patency survival A multivariate Cox proportional hazards regression analysis was performed to adjust for catheter insertion technique (laparoscopic vs percutaneous).Descriptive statistical approaches to compare outcomes in obese vs. non-obese were performed. Statistical analysis was performed using R version 4.2.2.

### Procedural details

All laparoscopic peritoneal dialysis catheter insertions were performed by renal transplant surgeons listed as authors (S’ON, JMcD and DMcG), while all percutaneous insertions were undertaken either jointly with, or independently by, a consultant interventional radiologist (DC); no nephrologist-only procedures were conducted in the Regional Nephrology and Transplant Centre during the study period. Laparoscopic PD catheter insertions were conducted in theatre under general anaesthesia. Following initial camera port access, the PD catheter was introduced under direct vision using either a 16Fr Peel-Away® Sheath Introducer (Cook Medical, Hitchin, UK) or an eight-millimetre laparoscopic port (Endopath® Xcel, Ethicon, London, UK, Ethicon Endo-Surgery). A rectus sheath tunnel was created to direct the catheter toward the pelvis [19]. All catheters used were coiled-tip designs. Additional laparoscopic ports were employed only in selected cases if adhesiolysis, omentopexy, catheter repositioning, or suture fixation was required.

Fluoroscopically guided percutaneous PD catheter insertions were performed in the interventional radiology suite at the Regional Centre with a consultant Interventional Radiologist present, utilising ultrasound to avoid injury to vessels and viscera, and fluoroscopy to confirm safe needle entry by tracking contrast flow around bowel loops. A guidewire was advanced towards the pelvis, and the PD catheter was introduced via a 16Fr Peel-Away® Sheath Introducer (Cook Medical) after dilating the tract.

At our centre, the choice between percutaneous and laparoscopic catheter insertion is guided by clinical factors including anaesthetic risk, prior abdominal surgical history, and anatomical considerations. In obese patients (BMI ≥ 30), laparoscopic insertion is generally preferred to facilitate accurate pelvic catheter placement, allow simultaneous hernia repair if necessary, and optimise the location of the catheter exit site through use of extension segments where needed. For all patients the following peri-procedural care was followed. Prior to the procedure, patients underwent decolonisation for Methicillin-resistant *Staphylococcus aureus* (MRSA) and Methicillin-sensitive *Staphylococcus aureus* (MSSA) and received bowel preparation. Intravenous Teicoplanin was administered for antibiotic prophylaxis. Post-insertion, PD catheters were subcutaneously tunnelled to the chosen exit site using a 16Fr drain spike from a high vacuum wound drainage system (Medinorm®, Summit Medical, Cotswolds, UK). Catheter extensions (Covidien Argyle, Adult Titanium Catheter Extender, Medtronic, Watford, UK) were used selectively in patients with large abdominal girth and abdominal wall mobility to facilitate exit sites in the upper abdomen and lower chest areas. Catheters were tested with Heparinised Saline (5000 units/500 mL) to ensure good inflow and outflow. Heparinised Saline was left in the peritoneal cavity to reduce the risk of omental wraps and fibrin plugs. Exit sites were dressed in a Biopatch (Ethicon), sterile gauze, and 3 M™ Tegaderm™ Transparent Film Dressings, which were left undisturbed for five days.

## Results

A total of 157 patients had a PD catheter insertion between 01/01/2020-31/12/2023, all were included in the analysis. 113 patients (72%) had a BMI < 30 and were classified as non-obese with a range of BMI of 16–29. 44 remaining patients (28%) were classified as obese with a BMI ≥ 30 range 30–48. The demographic details of patients categorised by BMI < 30 or BMI ≥ 30 can be found in Table 1.

**Table 1:** Demographics of patients undergoing laparoscopic or percutaneous peritoneal dialysis catheter insertions in 2020–23 (n = 157) by BMI

	Median (range) or N (%)		
Variable	BMI < 30 (n = 113)	BMI ≥ 30 (n = 44)	P value (Welch’s t-test)
Age	60 (17–88)	57 (18–83)	0.67
Sex			
Female	48 (42%)	18 (41%)	1.0
Male	65 (58%)	26 (59%)	1.0
BMI	25 (16–29)	33 (30–48)	-
Percutaneous	47 (42%)	9 (20%)	0.02
Laparoscopic	66 (58%)	35 (80%)	0.02
Primary renal disease			
Diabetic nephropathy	34 (30%)	14 (32%)	0.99
Polycystic kidney disease	15 (13%)	4 (9%)	0.65
IgA Nephropathy	6 (5%)	3 (7%)	1.00
Other	58 (51%)	23 (52%)	
Previous abdominal surgery (not laparotomy)	49 (43%)	20 (45%)	0.95
Previous laparotomy	10 (9%)	1 (3%)	0.27
Pre-procedure glomerular filtration rate	10 (4–22)	10 (5–14)	0.77
Failing transplant	11 (10%)	4 (10%)	1.0
Transfer from haemodialysis	16 (14%)	5 (11%)	0.84

There were no statistical differences between the non-obese and obese groups regarding age, gender, primary renal disease, previous surgery or modality transfer from HD or failing transplantation. However, catheter insertion technique did statistically differ between the two groups. 80% of obese patients had their PD catheter insertion via a laparoscopic approach compared to 58% of non-obese patients.

### PD catheter patency outcomes

**Fig. 1:**
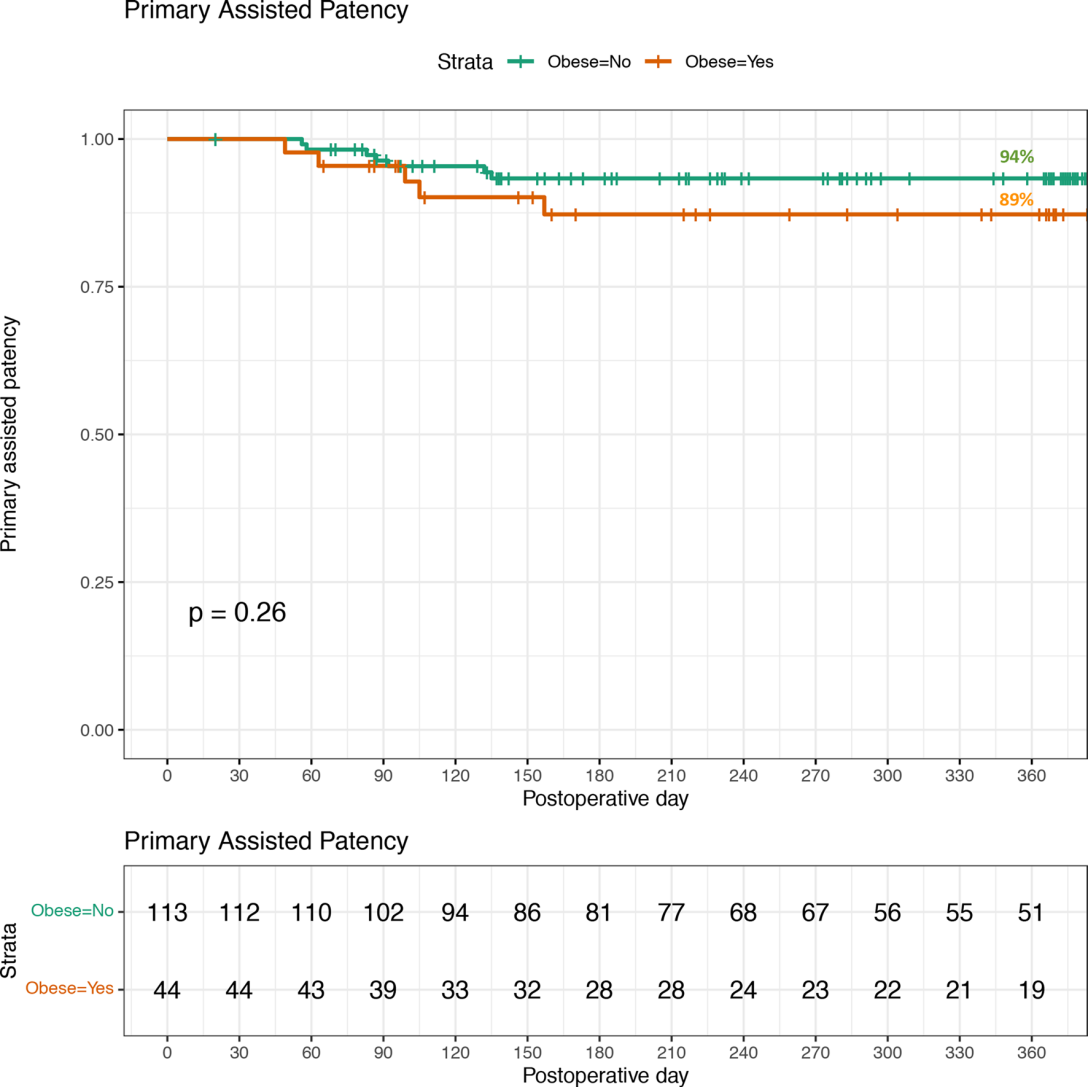
Primary patency for peritoneal dialysis catheter insertions in 2020-2023. Non-obese=113, Obese=44. Loss of patency is censored for death, transplant, infection, or transfers to HD because of inadequate dialysis, psychosocial reasons, or medical problems

Primary patency for peritoneal dialysis catheter insertions is demonstrated in Fig. 1. The primary patency rate for patients with a BMI < 30 was 88% (100/113) compared to 80% (35/44) in the cohort with a BMI ≥ 30, however the differences in results were not statistically significant (p = 0.13). Three patients with BMI of 39, 41 and 48 utilised extended PD catheters, all patients with extended catheters achieved primary patency. Stratified analysis showed that in patients with BMI < 30, primary patency rates were 89% following laparoscopic insertion and 87% following percutaneous insertion. In patients with BMI ≥ 30, primary patency rates were 74% following laparoscopic insertion and 100% following percutaneous insertion, although the small number of obese patients undergoing percutaneous insertion (n = 9) limits interpretation.

**Fig. 2:**
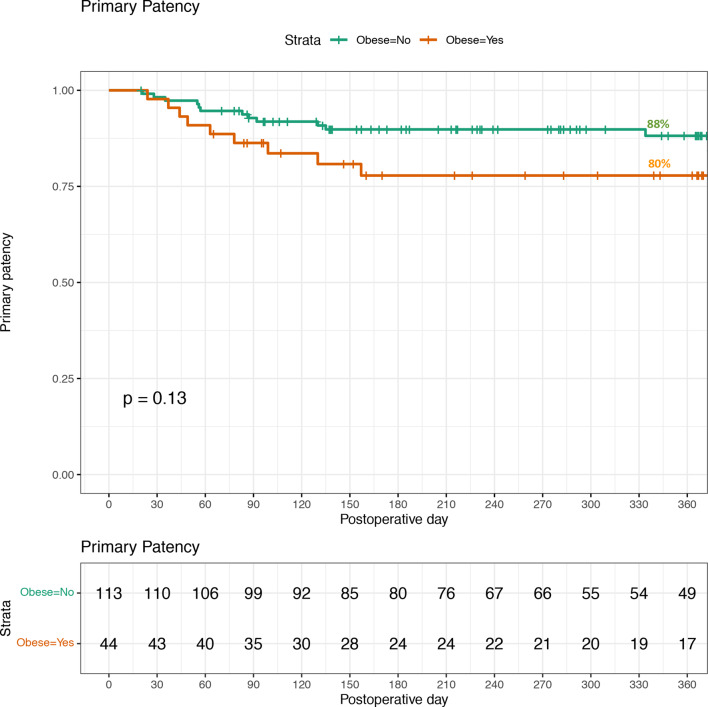
Primary assisted patency for peritoneal dialysis catheter insertions in 2020-2023. Non-obese=113, Obese=44. Loss of patency is censored for death, transplant, infection, or transfers to HD because of inadequate dialysis, psychosocial reasons, or medical problems

The primary assisted patency rates for both non-obese and obese individuals is displayed in Fig. 2. Primary assisted patency for those with a BMI < 30 was 94% (106/113) compared to 89% (39/44) in those with a BMI ≥ 30. The observed difference was statistically insignificant (p = 0.26).

**Fig. 3:**
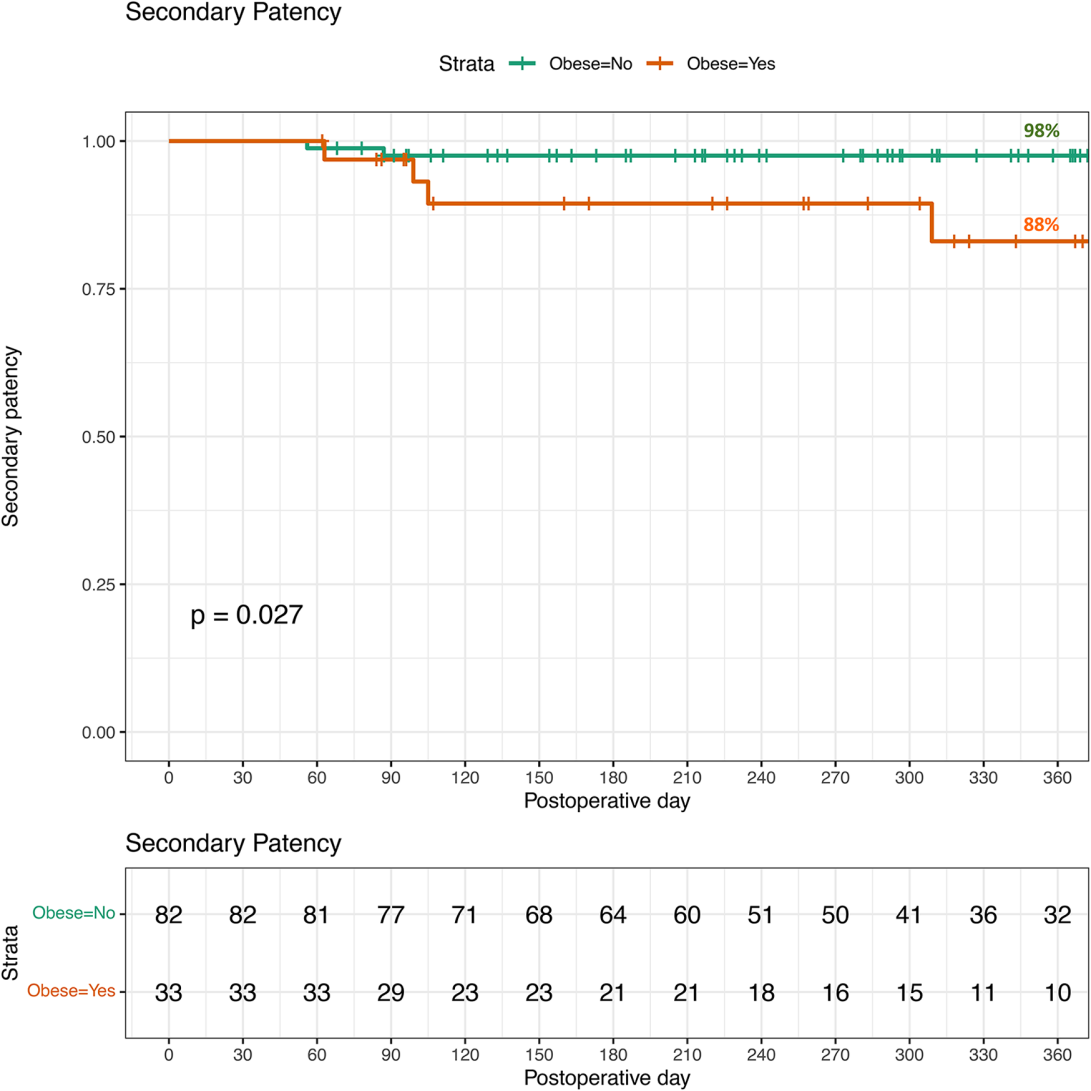
Secondary patency for peritoneal dialysis catheter insertions in 2020–2023. Non-obese=113, Obese=44. Loss of patency is censored for death, transplant, infection, or transfers to HD because of inadequate dialysis, psychosocial reasons, or medical problems

The secondary patency rates for both non-obese and obese individuals is displayed in Fig. 3. Four catheters in total were replaced (three in the BMI < 30 group, one in a patient with a BMI of 43). All four catheters were replaced due to flow dysfunction following conservative management and attempt at manipulation. Only two catheters functioned after replacement (both of which were in the BMI < 30 group). Secondary patency for those with a BMI < 30 was 96% (108/113) were not different compared to 89% (39/44) in those with a BMI ≥ 30 (p = 0.11).

The ISPD standard of > 80% 12-month patency was used as the benchmark across all catheter insertions (laparoscopic and percutaneous). For advanced laparoscopic techniques the benchmark is higher at > 95% and was not reached. Not all advanced laparoscopic techniques (e.g., omentopexy) were applied in all cases but rectus sheath tunnelling was routinely used. Further routine use of omentopexy may lead to higher patency rates, particularly in the obese group. After adjustment for insertion technique in a Cox regression model, BMI ≥ 30 kg/m² was not independently associated with loss of catheter patency (adjusted hazard ratio: HR 1.81, 95% CI 0.55–4.32, *p* = 0.18).

### PD Catheter associated infection outcomes

Exit site, tunnel infections and PD catheter associated peritonitis episodes 1-year post catheter insertion were collected for all 157 patients. To account for patients who did not complete the full one-year follow-up due to events such as transplantation, transfer to HD, or death, the infection rates were calculated based on the actual time at risk. Follow-up time was measured in months and converted to patient-years. Infection rates were then expressed as episodes per patient-year by dividing the total number of episodes by the sum of follow-up time across all patients.

A total of 28 exit site infection episodes were recorded, resulting in a rate of 0.23 episodes per patient-year. 6 episodes of tunnel infection were recorded, resulting in a rate of 0.07 episodes per patient-year. There were 33 episodes of catheter associated peritonitis, resulting in a rate of 0.31 episodes per patient-year. All observed rates were well within the ISPD guideline-recommended thresholds of ≤ 0.40 for exit site infections and ≤ 0.50 for PD catheter associated peritonitis.

The difference in infection rates between non-obese (BMI < 30) and obese (BMI ≥ 30) patients who underwent a PD catheter insertion is outlined in Table 2. Whilst none were statistically significant, there did appear to be a trend of higher rates of PD catheter associated peritonitis in obese patients.

**Table 2:** Comparison of infection episodes between non-obese and obese patients, 1 year follow-up post PD catheter insertion 2020–2023

Infection Episodes	Total Number	Number BMI < 30 (%)	Number BMI ≥ 30 (%)	P value (Welch’s t-test)
Exit Site Infection	28	16	12	0.19
Tunnel Infection	6	4	2	0.96
Catheter associated peritonitis	33	19	14	0.09

### Mortality and transfer to different kidney replacement therapy modality outcomes

The overall post catheter insertion 1-year outcomes for all 157 patients are detailed in Table 3. There were no significant differences in completing 1 year PD, transplantation, transfer to HD, withdrawal from dialysis or death between the non-obese or obese cohorts. However, there does appear to be a trend for higher rates of transfer to HD in the obese group (34% vs 19%, p = 0.06).

**Table 3:** Outcomes of patients undergoing laparoscopic or percutaneous peritoneal dialysis catheter insertions in 2020–23 (n = 157) by BMI

	N (%)		
Outcome	BMI < 30 (n = 113)	BMI ≥ 30 (n = 44)	P value (Welch’s t-test)
Completed 1-year follow up on PD	52 (46%)	18 (41%)	0.69
Transplanted	29 (26%)	7 (16%)	0.27
Transfer to HD	21 (19%)	15 (34%)	0.06
Withdrawal from dialysis	1 (1%)	0 (0%)	1.0
Died	10 (9%)	4 (6%)	1.00

Transfer of modality to HD occurred in 21 patients in the non-obese group due to; flow dysfunction (n = 5) pleural leak (n = 4), psychosocial reasons (n = 4), inadequacy (n = 3), PD peritonitis (n = 2), tunnel infection (n = 2), and drain pain (n = 1). There were 15 patients in the obese cohort who transferred to HD within 1-year post PD catheter insertion. The reasons for transfer were flow dysfunction (n = 5), PD peritonitis (n = 4), scrotal leak (n = 2), inadequacy (n = 1), tunnel infection (n = 1), psychosocial (n = 1) and diverticular perforation (n = 1).

The reasons for death 1-year post PD catheter insertion in the non-obese group (n = 10) were unknown (day 142), stroke (day 213), gastrointestinal perforation (day 242), ischemic colitis (day 291), multi-organ failure (day 273), sepsis (day 275), myocardial infarction (day 275), cardiac failure (day 297), Covid (day 309) and diabetic ketoacidosis (day 348). In the obese cohort the reason for death (n = 4) were multi-organ failure (day 65), pneumonia (day 95), myocardial infarction (day 220) and arrhythmia (day 363). There was no associated perioperative mortality for either group.

## Discussion

Historically obesity was seen as a relative contraindication to commencing PD. This study sought to compare patency, infection, dialysis modality transfer and mortality in both non-obese and obese patients in a single region in the United Kingdom. It demonstrated both groups achieved acceptable patency rates according to ISPD benchmarks with no significant difference between obese and non-obese. Infective complications were also with ISPD guidelines, though there was a non-significant trend towards higher catheter associated peritonitis rates in obese patients. Finally, there was a non-significant trend towards a higher rate of modality transfer to HD in the obese cohort. We intend to explore this trend with a longer follow-up observation period, i.e. 2-year follow-up.

### Patency and catheter placement techniques

Whilst the literature regarding optimal PD catheter placement in the obese is small, our findings corroborate with previously published studies regarding patency. Krezalek et al. have reported that in their study comparing 70 obese patients vs 161 non-obese patients; obesity did not increase complications or shorten dysfunction-free PD catheter survival regardless of operative technique (open, basic laparoscopic or advanced laparoscopic) [22]. Other studies have demonstrated that percutaneous insertion of PD catheters is safe irrespective of BMI, however total number of obese patients were small, and cut-off for being labelled obese was a BMI ≥ 28 [23].

The use of extended PD catheters can further optimise catheter position in obese patients, offering the ability to position the exit site remotely, away from problematic and mobile lower abdominal regions. Whilst only used in a small number of cases in this cohort, it was highly effective in those with an extremely high BMI and a particularly unfavourable abdominal wall pannus. A United States based anthropometric study of an adult population measured and highlighted that fewer than 20% of patients with BMIs between 35 and 39.9 could achieve an ideal balance of pelvic catheter tip positioning and suitable exit site location with standard abdominal catheters [24]. Furthermore, no individuals with BMIs exceeding 40 could be adequately fitted with standard catheters. Supporting this, another study compared outcomes for patients using extended catheters (average BMI ≥ 33.6) to those using standard abdominal catheters (average BMI 26.9). Results showed that the survival time until the first exit site infection was significantly longer for the extended catheter group. Additionally, there was a noted trend towards lower infection rates with extended catheters placed at non-lower abdominal exit sites [25]. Whilst numbers of extended catheters are small in our cohort, three patients with BMI of 39, 41 and 48, all achieved primary patency and none had infective complications, hence we are adopting their use further into our practice.

We acknowledge that surgical technique was not evenly distributed between groups and may influence outcomes. Laparoscopic insertion, known to improve catheter survival through advanced placement techniques such as rectus sheath tunnelling, was more commonly utilised in obese patients. Multivariate adjustment for insertion technique, however, confirmed that obesity itself was not a significant predictor of loss of catheter patency. This highlights that with appropriate surgical strategies, patients with obesity can achieve comparable PD outcomes to non-obese patients. The higher rates for laparoscopic insertion in obese patients is supported by previously published studies suggesting that advanced laparoscopic techniques, including rectus sheath tunnelling and selective omentopexy, are effective in reducing catheter dysfunction, even in patients with prior abdominal surgery [11–13].

### Infective complications in patients with obesity

Whilst some studies have suggested obesity does not increase the risk of infective complications [23, 25] The observed trend toward higher peritonitis rates in obese patients in this study is consistent with findings by McDonald et al., who noted that obesity is associated with worse peritoneal dialysis outcomes, including higher infection rates, in the Australia and New Zealand patient populations [26]. This was also seen in a Japanese study were a BMI ≥ 25 at time of catheter insertion was associated with Hazard Ratio of 2.08 for developing peritonitis [27].

### Dialysis modality transfer to HD

The higher, though not statistically significant, rate of transfer to HD among obese patients in our study mirrors concerns raised in the literature about the challenges of maintaining PD in this population [27]. However, studies have also shown that with appropriate management, obese patients can achieve comparable survival outcomes on PD [9, 28].

### Limitations, implications for practice and future research directions

This study is limited by its single-region design and small team of operators. This is not unique within this area of the literature and in fact this study has equitable if not larger cohorts than other recently published studies with an obesity BMI cut-off of ≥ 30 [23, 27, 28]. This study’s sample size may limit the detection of small between-group differences. Notably, our cohort is as large as or larger than those of similar studies. A post-hoc calculation suggests that detecting an 8% absolute difference in 1-year patency with 80% power would require over 300 patients per group, far exceeding our sample. Thus, the absence of statistically significant differences should be interpreted with caution, as the study may be underpowered to detect modest effect sizes. The study like most others within the literature is limited by the use of BMI as a measure of obesity. Central obesity and waist circumference are likely to be more useful metrics to determine catheter malfunction and infective risks and merits further research. This study is also limited by the lack of systematic laboratory data, comorbidity profiles, and nutritional assessments at the time of catheter insertion, which may confound outcomes but were not available for inclusion. Future prospective studies should aim to collect these data to enable more comprehensive risk adjustment. This study contributes to the growing evidence that obesity should not be considered a contraindication for PD. With advanced surgical techniques and comprehensive perioperative care, obese patients can achieve outcomes comparable to their non-obese counterparts. This supports the expansion of PD programs to include obese patients, addressing the increasing prevalence of obesity in the ESKD population. This may be of particular interest to those working in resource limited systems where financial barriers to more expensive treatment modalities than PD such as HD can be limited. Although a formal economic analysis was not conducted, peritoneal dialysis (PD) is widely recognised as more cost-effective than haemodialysis (HD), including in resource-limited settings [15]. The clinical viability of PD in obese patients, as demonstrated in this study, suggests that the general economic advantages of PD extend to this population [9]. Furthermore, PD expansion strategies may be particularly valuable in reducing dialysis-related healthcare costs and improving access to kidney replacement therapy [14].

The evidence base for PD in obesity could be improved using larger prospective multicentre trials to validate safety outcomes whilst being adequately powered to fully investigate trends that we have reported that were found to be insignificant. Evaluating advanced surgical techniques, such as specific laparoscopic methods and the use of extended catheters in obese patients, is essential to optimise PD outcomes. Additionally, assessing the long-term impact of obesity on PD success, quality of life, and healthcare costs will provide a comprehensive understanding of treatment efficacy. Finally, a better understanding of the role of bariatric surgery or glucagon-like peptide-1 (GLP-1) receptor agonists in optimising patients for kidney replacement therapy remains relatively unexplored.

## Conclusions

Despite historical hesitancy, this study demonstrates that obese patients can achieve comparable PD outcomes to non-obese individuals. These findings challenge biases against offering PD to obese patients and support the expansion of PD programs to accommodate this growing population, ensuring equitable access to kidney replacement therapies.

## Electronic supplementary material

Below is the link to the electronic supplementary material.

Supplementary Material 1

## Data Availability

The datasets generated analysed during the current study are not publicly available due their clinical nature and patient confidentiality but are available from the corresponding author on reasonable request.
